# The Effect of a Patient Navigator on Treatment Abandonment and Follow-up for High Grade Osteosarcoma Patients in the Philippine General Hospital

**DOI:** 10.31557/APJCP.2021.22.9.2873

**Published:** 2021-09

**Authors:** Czar Louie Gaston, Kathleen Taleon, Ken Bersales, Cesar Dimayuga, Jochrys Estanislao, Pamela Fajardo, Albert Quintos, Donnel Rubio, Edward Wang, Ana Patricia Alcasabas

**Affiliations:** 1 *Department of Orthopedics, College of Medicine and Philippine General Hospital, University of the Philippines Manila. *; 2 *Department of Pediatrics, College of Medicine and Philippine General Hospital, University of the Philippines Manila. *

**Keywords:** Osteosarcoma, abandonment, navigator, LMIC (Low to middle income country)

## Abstract

**Introduction:**

Treatment abandonment for osteosarcoma is a significant problem in developing countries with rates as high as 70%. This study aimed to determine the effect of a patient navigator on treatment abandonment and patient follow-up of osteosarcoma patients at a tertiary referral center.

**Materials and Methods:**

A retrospective review of osteosarcoma patients was performed investigating 2 cohorts based on the start of the patient navigator. Group 1 (Pre-Patient Navigator, n=46) were treated from January 2016 to December 2017 while Group 2 (Post-Patient Navigator, n=29) were treated from January 2018 to June 2019. The primary outcome investigated was treatment abandonment defined as missing 4 or more consecutive weeks of treatment. Semi-structured interviews were conducted to investigate the effect of the patient navigator on the cohorts.

**Results:**

Treatment abandonment rates for the Pre-Patient Navigator group was significantly higher compared to those with a patient navigator (50% vs 6%, p=0.0001). Abandonment for the pre-navigator group occurred at a mean of 2.9 months (0 – 9 months, median 3 months). Fourteen of 23 patients who abandoned from Group 1 did not proceed to neoadjuvant chemotherapy while 3 patients abandoned after completing 1 cycle of neoadjuvant chemotherapy. In the patient navigator group, no patients abandoned prior to completing 3 cycles of chemotherapy. One patient abandoned after refusing a below knee amputation after 3 cycles of neoadjuvant chemotherapy and 1 patient did not complete further chemotherapy after having a hip disarticulation. Patient feedback on the patient navigator experience was favorable.

**Conclusions:**

Having a patient navigator from diagnosis throughout treatment reduced treatment abandonment rates in osteosarcoma patients and may serve as a model for other low to middle income countries.

## Introduction

Conventional high-grade osteosarcoma is the most common primary bone sarcoma in children with an estimated incidence in the Philippines of 200 – 300 cases/year. Standard treatment consists of a combination of multi-agent chemotherapy given for 6 months and surgery for local tumor control. Survival rates for osteosarcoma treated by dedicated sarcoma units range from 80-90% 2-year and 50-70% 5-year over-all survival. In contrast, survival rates in the Philippines are dismal with 2-year over-all survival of 50% and treatment abandonment rates up to 36%, which is similar to other low-and-middle income countries (Choeyprasert et al., 2013; Friedrich et al., 2013; Noor et al., 2014; Monsereenusorn et al., 2019). Failure of coordination of the complex treatment strategies required for osteosarcomas has been identified as a risk factor for treatment abandonment (Rivera et al., 2008; Friedrich et al., 2013).

Patient navigators are trained personnel who guide patients through the healthcare system from diagnosis, treatment, and follow-up. They perform different tasks such as patient and family education, exploring transport options to treatment centers, coordinating diagnostic and treatment schedules, personal communication and encouragement to complete treatment (Friedrich et al., 2013). 

Navigators have been shown to improve adherence to medication, completion of treatment, and reduce follow-up loss in chronic diseases requiring complex and prolonged treatment such as hemoglobinopathies, kidney transplant, and HIV (Sullivan et al., 2012; Mizuno et al., 2018; Allemang et al., 2019). Navigators have also been used for improving delivery of care in various cancers including lung, prostate, colorectal, and breast (Freeman et al., 1995; Fiscella et al., 2012; Wagner et al., 2014, dela Rama and Pratz, 2015; Zibrik et al., 2016; DeGroff et al., 2017).

In an attempt to decrease treatment abandonment, a research nurse assistant has been performing patient navigation duties for osteosarcoma patients in the Philippine General Hospital since January 2018. The role includes 1). Establishing and maintaining communication pathways through mobile phone and social media applications, 2). Organizing scheduled diagnostic and treatment appointments, 3). Coordinating multidisciplinary team input, 4). Facilitating family meetings with sponsors for healthcare funding and lodging, and 5). Forming social bonds with the patient and their families. 

This study aimed to determine the effect of a patient navigator on osteosarcoma treatment abandonment and patient follow-up at the Philippine General Hospital. We hypothesized that abandonment rates decreased with the introduction of the patient navigator in 2018. The results of this study will be used to guide improve changes in the osteosarcoma patient pathway at the Philippine General Hospital to decrease treatment abandonment, increase treatment compliance, and improve cancer outcomes.

## Materials and Methods

A retrospective chart review of high-grade osteosarcoma patients treated at the Philippine General Hospital from January 2016 to June 2019 was performed. Patients for inclusion in the study included all high-grade osteosarcoma patients confirmed by histopathology review by the Department of Pathology of the Philippine General Hospital, diagnosed and treated at the outpatient clinic of the Philippine General Hospital. Patients who were seen for slide review and opinion only, who did not receive any treatment, were excluded from the study.

The population was divided into 2 cohorts based on the starting time point of a patient navigator. Group 1 (Pre-Patient Navigator) were treated from January 2016 to December 2017, before the start of the patient navigator while Group 2 (Post-Patient Navigator) were treated from January 2018 to June 2019, after the start of the patient navigator. 

A standard osteosarcoma treatment protocol was followed for the treatment of osteosarcoma patients during the whole study period with no changes between the 2 groups. Treatment routinely consisted of 6 cycles of Adriamycin and Cisplatin with surgery after 3 chemotherapy cycles, either amputation or limb salvage depending on the extent of the tumor. Primary amputation was offered in cases with large, fungating, or bleeding tumors. 

The primary outcome investigated was treatment abandonment, defined as missing 4 or more consecutive weeks of treatment (Fiscella et al, 2012). Refusal of treatment (chemotherapy or surgery) after being diagnosed with osteosarcoma was also counted as treatment abandonment. 

Outpatient clinic follow-up after treatment (completion of chemotherapy) was also investigated for the 2 cohorts. Lost to follow-up was defined as missing 2 or more consecutive outpatient follow-up appointments.

To investigate the effect of the patient navigator on the cohorts, interviews with questions relating to the patient’s and parent’s experience with the sarcoma navigator were conducted. Patients and their parents of the 2 groups were identified during their scheduled outpatient follow-up where informed consent for the interview was obtained by the investigators [CLG, KT, KB] using written informed consent forms, verbal assent forms for patients 7 – 12 years of age, and simplified assent forms for patients aged 12 – 15. 

A semi-structured interview guide was used which included questions that explored the patient’s and parent’s experience with the sarcoma navigator only and did not deal with specifics of their treatment or outcomes which may be disturbing or distressing to the patients. Only patients from Group 2 had to answer questions that pertained to the effects of the patient navigator during active treatment (diagnosis, chemotherapy and surgery). 

The interviews were conducted by the co-investigators [KT, KB] in a private room in the PGH outpatient clinics. The interviews were recorded verbatim on an encrypted tablet, transcribed, and reviewed. The responses were tabulated and recurring themes were emphasized. To avoid coercion and bias, the sarcoma navigator was not present during the consent process and interview. 

Statistical analysis was performed on Statview version 5.0.1, SAS Institute Inc. NC, US; 1998. Demographics, treatment abandonment rates and follow-up rates were compared between groups by chi-square and t-test with a p<0.05 considered significant. Variables associated with treatment abandonment on univariate analysis (p<0.1) were entered into a multiple logistic regression model to assess for independent risk factors for treatment abandonment in osteosarcoma patients.

This study was approved by the University of the Philippines Manila REB and received grant funding from the UPM-PGH Expanded Hospital Research Office.

## Results

A total of 75 patients were included in the study, Group 1 (Pre-Patient Navigator, n=46) treated from January 2016 to December 2017 and Group 2 (Post-Patient Navigator, n=29) treated from January 2018 to June 2019. The demographic profiles of the 2 groups were comparable (Table 1).

Patients in the navigator group had significantly lower treatment abandonment compared to the pre-patient navigator group (6.8 vs 50%, Table 2). None of the patients in the navigator group have discontinued follow-up contrasted to 45% prior to the patient navigator.

In the pre-navigator group, abandonment occurred at a mean of 2.9 months (0 – 9 months, median 3 months). 60% of Group 1 patients (n=14/23) did not proceed to neoadjuvant chemotherapy while 3 patients abandoned after completing 1 cycle of neoadjuvant chemotherapy. Four patients abandoned after undergoing surgery (amputation) and did not proceed with completion of adjuvant chemotherapy. In 2 patients, abandonment occurred after disease progression during chemotherapy. 

In the patient navigator group, no patients abandoned prior to completing 3 cycles of chemotherapy. One patient abandoned after refusing a below knee amputation after 3 cycles of neoadjuvant chemotherapy and 1 patient did not complete further chemotherapy after having a hip disarticulation. 

A subgroup analysis of localized osteosarcoma, excluding those with metastatic disease at diagnosis, showed that pre-navigator Group 1 had 21% abandonment compared to 7% in the patient navigator group.

No patient navigator (OR 17) remained an independent risk factor to abandonment in a multiple logistic regression model. Other independent risk factors determined in this study were male sex (OR 3.9) and advanced disease on diagnosis (OR 4.6) (Table 3).

16 patients on active follow-up consented to the interview portion of the study. Majority were introduced to the patient navigator at the outpatient clinics. Two patients were first seen in the in-patient setting (emergency room, ward) after being admitted on initial presentation with large, bleeding and fungating tumors. Three patients already on active outpatient follow-up first met the patient navigator in an organized social meeting (annual graduation party) for osteosarcoma patients of the department. 

Common themes regarding effect of patient navigator during diagnosis, treatment, and follow-up include giving a cell phone number that parents could get in touch with, explaining about the disease and treatment, giving reminders about treatment schedules and follow-up, and helping procure medication and surgical implants. All the patients interviewed gave positive feedback regarding their patient navigator experience (Appendix 1).

**Table 1:** Profile of Osteosarcoma Patients in Philippine General Hospital from 2016 – 2019

	Group 1	Group 2	p-value
	(Pre-patient Navigator) n= 46	(Patient Navigator) n=29	
Sex			
Male	65% (30/46)	69% (20/29)	0.96
Age	14.8 years	14.2 years	0.3
Metastases at diagnosis			
Yes	69% (32/46)	55% (16/29)	0.22
Surgery			
Amputation	79% (22/28)	60% (15/25)	0.14

**Table 2:** Treatment Abandonment and Follow-up of Osteosarcoma Patients in Philippine General Hospital from 2016 – 2019

	Treatment Abandonment	p-value	Lost to Follow-up	p-value
Patient Navigator				
No (Group 1)	50% (23/46)	0.0001	45% (5/11)	0.01
Yes (Group 2)	6.8% (2/29)		0 (0/16)	
Sex				
Male	40% (20/50)	0.08	22% (4/18)	0.24
Female	20% (5/25)		7.1% (1/14)	
Metastases at diagnosis				
Yes	43% (21/48)	0.01	28% (4/14)	0.07
No	14% (4/27)		5.5% (1/18)	
Surgery				
Amputation	16% (6/37)	0.32	21% (4/19)	0.34
Limb salvage	6.2% (1/16)		8.3% (1/12)	

**Table 3:** Multiple Logistic Regression Model for Risk Factors for Treatment Abandonment of Osteosarcoma Patients in Philippine General Hospital from 2016 – 2019

R^2^ = 0.285	Odds Ratio (95% CI)	p-value
Patient Navigator		
No	OR 17.2 (3 – 90)	0.0007
Sex		
Male	OR 3.9 (1 – 14)	0.04
Metastases at diagnosis		
Yes	OR 4.6 (1.1 – 18)	0.027

OR, Odds ratio; CI, Confidence Interval

## Discussion

Osteosarcomas require complex multidisciplinary management from different medical specialties for appropriate diagnosis and treatment. Consequently, treatment abandonment, especially in resource challenged countries, is a significant concern (Choeyprasert et al., 2013; Friedrich et al., 2013; Noor et al., 2014; Monsereenusorn et al., 2019). As documented in the present study, abandonment rates in the PGH setting were distressingly high and recognizing this, a patient navigator was put in place to try and improve treatment abandonment. 

Navigators have been used to improve treatment compliance and completion in different diseases including cancer and our experience validates this in a Philippine setting. Starting in 2018, abandonment rates of osteosarcoma patients in our unit have significantly decreased and this coincides with the start of our osteosarcoma patient navigator.

In the pre-navigator group, a large majority of those who abandoned did so in the early stages of treatment, many receiving 1 cycle of chemotherapy or less. Friedrich et al reported similar findings in their study where abandonment for osteosarcoma patients was common in the first 3 months after diagnosis (Friedrich et al., 2013). For this, they cite avoidance of amputation as a possible reason for abandonment which was also seen in our study. 

For diagnosis and treatment, osteosarcoma patients in PGH go through multiple appointments, in different departments and areas of the hospital. Treatment abandonment even before starting chemotherapy suggests a confusing and frustrating experience for patients. Through interviews, patients express gratitude that the navigator “gave them directions through the hospital” and “guided them step-by-step until finishing treatment”. Part of the navigator’s duties include organizing diagnostic and treatment appointments so that patients completed multiple scans and specialist evaluations in 1 or 2 hospital visits and since starting, no patient has abandoned without at least completing 3 cycles of neoadjuvant chemotherapy.

Other risk factors for abandonment seen in the study were advanced disease (metastases at diagnosis) and male sex. Treatment abandonment is a complex process affected by factors such as family dynamics, public disease perception, and cultural views (Mostert et al., 2011; Friedrich et al., 2016). Maintaining good communication is vital and patient navigators can act as a bridge between clinicians and patients and their families, guiding and supporting them outside of the clinic setting, when they have had more time to reflect on the information given to them. “Explaining the disease and treatment” was a common positive trait of the navigator as cited by patients in the interviews. Additional training in disciplines such as psychiatry and enhanced communication may help navigators detect difficulty in coping and identify patients at risk of abandonment that need intervention (Kashima et al., 2018).

Several studies support patient navigators being part of cancer care in resource limited settings. A retrospective study in one of the medical centers in Tanzania showed significant impact of patient navigators in patient outcomes of pediatric cancers through reduced time to evaluation and diagnosis from 49.7 days to 16.6 days (p = 0.02) as well as in decreased rate of abandonment 50% to 38% (p < 0.001) (Alvi et al., 2017). A local study involving breast cancer patients noted a decreased attrition rate 62% to 18% (p<0.0001) and improved quality of life of breast cancer patients after navigation program was employed (Patdu et al., 2015). Another impact was seen in the field of retinoblastoma wherein employing a navigator led to earlier detection of the disease thereby increasing the census by 40%, and abandonment and refusal to treatment also decreased significantly from 52% to 11% (Alcasabas et al., 2017). These, among other studies, proved the importance of patient navigators as a strategy in improving cancer treatment outcome even in the low-to middle income country setting.

The effect of the osteosarcoma patient navigator in our institution continued into the time of COVID-19. Even with limited access to the hospital due to the SARS-COV2 pandemic, patients continued their treatment and follow-up through online platforms with the aid of the navigator (Gaston et al., 2020).

A limitation of this study is the majority of the patient population had metastatic disease on diagnosis. However, the treatment rendered at diagnosis was the same for both localized and advanced disease in both pre-navigator and navigator groups. Due to the design of the study, there was less time for follow-up for the navigator group and more patients may abandon treatment given longer follow-up. However, the decrease in early abandonment (<3 months) compared with the pre-navigator group is clearly evident and is a good indicator of the success of the intervention. 

In conclusion, having a patient navigator from diagnosis throughout treatment reduced treatment abandonment rates in osteosarcoma patients in a low to middle income country setting and may serve as a model to decrease abandonment for other developing countries. Further studies are planned to study the long-term effect of the patient navigator and replicate this in other hospitals in the Philippines. 

## Author Contribution Statement

CLG: Concept of study, wrote protocol and manuscript, collected data, analyzed data, reviewed and approved final manuscript; KT: Concept of study, collected data, reviewed and approved final manuscript; KB: Concept of study, collected data, reviewed and approved final manuscript; CD: Concept of study, reviewed and approved final manuscript; JE: Concept of study, reviewed and approved final manuscript; PF: Concept of study, reviewed and approved final manuscript; AQ: Concept of study, reviewed and approved final manuscript; DR: Concept of study, reviewed and approved final manuscript; EW: Concept of study, reviewed and approved final manuscript; APA: Concept of study, analyzed data, reviewed and approved final manuscript. Request for further data to be addressed to corresponding author (CLG) and subject to approval of UPM REB

## Conflict of Interests

The authors declare no conflict of interest
